# Heat Pump Technology in the Field of Fruit and Vegetable Drying: A Review

**DOI:** 10.3390/foods14152569

**Published:** 2025-07-22

**Authors:** Lichun Zhu, Xinyu Ji, Hao Yang, Xinze Cao, Wenchao Wang, Mengke Liang, Jiapin Li, Qian Zhang, Xuhai Yang, Zhihua Geng

**Affiliations:** 1 College of Mechanical and Electrical Engineering, Shihezi University, Shihezi 832003, China; zhulichun0204@126.com (L.Z.); 20211012426@stu.shzu.edu.cn (X.J.); 20221009152@stu.shzu.edu.cn (H.Y.); 20241009188@stu.shzu.edu.cn (X.C.); 20241009190@stu.shzu.edu.cn (W.W.); 20241009189@stu.shzu.edu.cn (M.L.); 20241009184@stu.shzu.edu.cn (J.L.); yxh_513@shzu.edu.cn (X.Y.); 2 Engineering Research Center for Production Mechanization of Oasis Special Economic Crop, Ministry of Education, Shihezi 832003, China; 3 Xinjiang Production and Construction Corps Key Laboratory of Modern Agricultural Machinery, Shihezi 832003, China

**Keywords:** heat pump technology, drying, combined heat pump technology, fruits and vegetables

## Abstract

Single or combined heat pump technologies are generally used to dry fruits and vegetables, with combined heat pump technologies offering superior performance. This review summarizes the applications of single and combined heat pump drying technologies for fruits and vegetables in China and globally, discusses their current advantages and disadvantages, and outlines future development directions for heat pump-based drying methods. Future research should focus on improving combined heat pump technologies and enhancing the performance of single heat pump drying systems to enhance the effectiveness and feasibility of these technologies for drying fruits and vegetables. Improved technologies would also help meet the increasing demand for high-quality food and social development. Moreover, changes in the mechanisms of key indicators, such as mechanical and thermodynamic properties, should be continuously monitored while drying various fruits and vegetables. Future research into combined heat pump technologies should focus on determining the conversion methods between pairs of drying technologies and identifying the most effective drying technology combinations. Future research into single heat pump technologies should focus on improving the performance levels of core components, such as compressors and drying media.

## 1. Introduction

**Figure 1:**
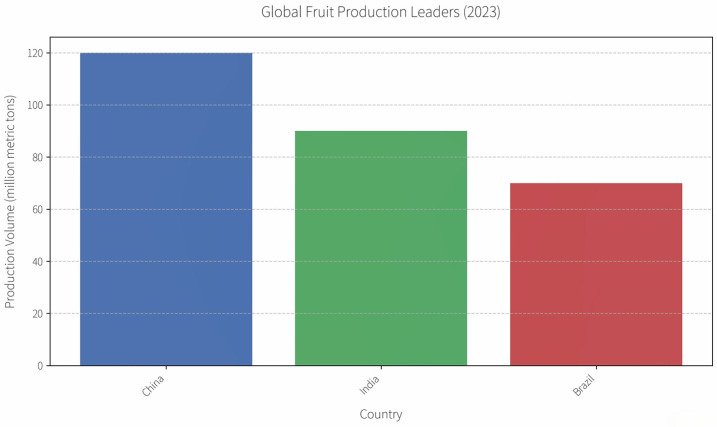
Global fruit production leaders (2023).

**Figure 2:**
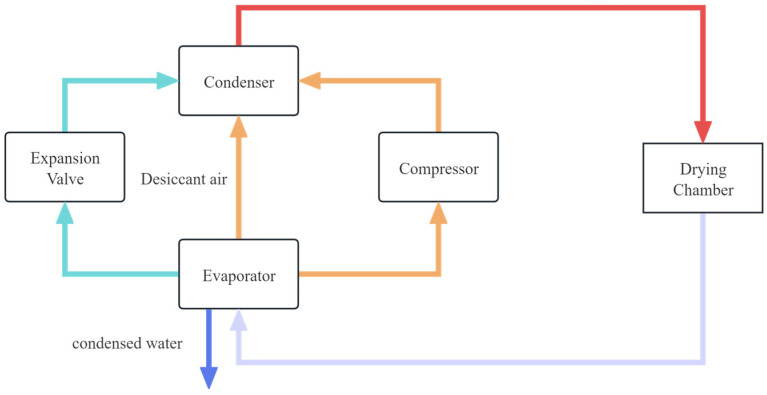
Heat pump dryness principle.

China is a major producer of fruits and vegetables (Figure 1), with an annual yield of 299.7 million tons of fruits [1]. However, the annual economic losses caused by fruit and vegetable corruption reached 13.94 billion dollars in 2024 [2]. Fruit and vegetable drying is a significant part of the food drying industry because it effectively extends the shelf life of fruits and vegetables, which enhances product value and increases farmers’ income. The main objectives of food drying are to preserve food by reducing water activity, decrease weight and volume, transform food into a more convenient form for storage, packaging, transportation, and/or use, and provide food with certain desirable characteristics, such as a distinct flavor, crunchiness, chewiness, among others [3]. The drying process of food products prevents microbial contamination and chemical changes such as enzymatic and non-enzymatic browning, extends storage life, and reduces packaging, handling, and transportation costs [4]. Among the various food drying technologies available, heat pump drying is widely used. Heat pump technology is considered to be a promising method for drying materials due to its high drying efficiency, high specific water extraction rate, and low investment cost [5]. Its fundamental principle is based on the reverse Carnot cycle, in which a small amount of electric energy is used to transfer heat from a low-temperature heat source to a high-temperature heat source. As hot air passes through the drying chamber, it absorbs moisture from the material, resulting in humidified air (Figure 2). This hot and humid air then enters the evaporator, where the refrigerant absorbs its heat and undergoes a phase change from liquid to gas. Consequently, the hot and humid air is cooled to its dew point, and moisture is condensed and removed. The gaseous refrigerant is compressed by the compressor and subsequently condensed back into a liquid phase. The heat released from this process reheats the air, which is then recirculated back into the drying chamber for continuous recycling [6].

The common shapes of dried fruits and vegetables include wet solids, slices, strips, and blocks. The water distribution within these products is complex and characterized by anisotropy and a multi-solid system. This complexity affects the drying behavior and final quality; thus, the quality of dried fruits and vegetables is generally evaluated based on their rehydration rate, nutrient retention rate, color, and taste [7]. Notably, the price of dried fruits and vegetables can increase up to 3.8 times their pre-drying value. At present, the demand for high-quality dried fruits and vegetables in the market is growing, maintaining good sensory quality and high nutritional content [4]. In China, the drying ratio is only 10%, which is significantly lower than that of developed countries such as the United States and Japan [8]. Effective drying technologies that ensure high quality, rapid drying speeds, low energy consumption, and ease of implementation for drying fruits and vegetables are urgently required in China.

This review summarizes the domestic and global application of single and combined heat pump drying technologies for fruits and vegetables, discusses their current advantages and disadvantages, and summarizes the future direction of heat pump technologies. A systematic literature review was conducted by comprehensively searching all records from the inception of each database to the present. The following English databases were interrogated: Web of Science Core Collection, Scopus, PubMed, and EBSCOhost (CAB Abstracts and FSTA included). Chinese databases comprised CNKI and Wanfang Data. Additional web-based sources were ProQuest Dissertations and Theses Global, OpenGrey, and Google Scholar. In each source, the Boolean search string (“heat pump” OR “heat-pump” OR “HPD” OR “refrigeration drying”) was applied. After deduplication, titles and abstracts were screened for relevance, followed by full-text evaluation for final inclusion. Iterative, temporally stratified syntheses were performed to ensure both rigor and comprehensive coverage across all periods.

## 2. Single-Stage Heat Pump Drying Technologies

Single heat pump drying technologies for fruits and vegetables are well established. Most available systems employ closed or semi-closed circulation drying chambers, which retain a significant portion of the original flavor profile of the agricultural product. The drying process can be divided into two types: direct drying and indirect drying. In the direct type, hot air or inert gas is used as the heat exchange medium. This method usually has a high water extraction rate [9]. Indirect drying utilizes surface heat exchangers to prevent direct contact between heat transfer media and materials. Compared to direct drying, this method has multiple advantages, including reducing contamination of the heat transfer medium, lowering the risk of combustion and explosion, and suppressing odors [10]. Indirect drying typically takes longer, and its efficiency is influenced by changes in material viscosity and volume shrinkage [11]. Compared with natural sun drying, these technologies are less susceptible to external environmental factors [12] and have superior drying efficiencies and development potential.

### 2.1. Current Status of Single Heat Pump Drying Technologies

**Figure 3:**
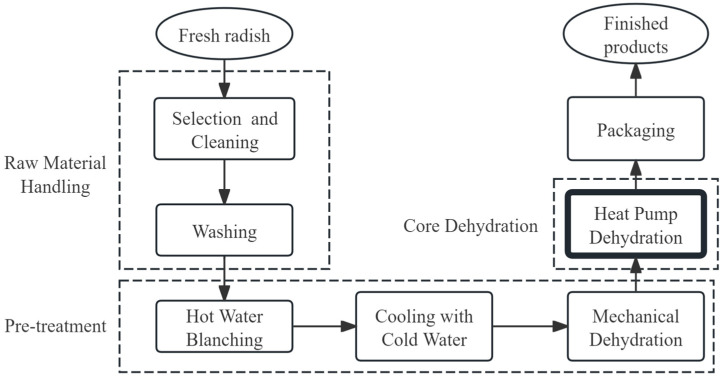
Flowchart of integrated heat pump drying process for white radish.

Single heat pump drying technologies create relatively low-temperature and controlled-humidity environments within drying chambers. They are able to dry heat-sensitive fruits and vegetables because the moisture migration rate on the surfaces of such fruits and vegetables reflects their internal mobility rate. Single heat pump drying also uses less energy consumption than traditional coal-fired drying, is a more convenient method for temperature control, and offers a high degree of automation. For example, dried radish, which retains a high nutritional value [13], can be processed using a single heat pump as follows (Figure 3):

Although extensive research has been conducted on compressors and refrigerants, drying processes must still be evaluated for various fruits and vegetables to establish accurate mathematical models. For many agricultural products, heat pump drying control systems require improvements before large-scale production can be implemented. A pilot study on drying shiitake mushrooms in Qingyuan County [14] suggested that implementing heat pump drying technology instead of traditional methods could save up to 40 million annually. Domestic single heat pump technologies have also been used for red dates, lemon slices, jackfruit, and mango slices [15]. These studies demonstrate that single heat pump drying technologies are superior to hot air-drying systems regarding the rehydration ratio, nutrient retention, maintenance cost, and service life. Moreover, single heat pumps for drying fruits and vegetables can be automated to a certain level [16], and many temperature and humidity sensors are compatible with heat pump drying systems [17]. Single heat pump drying systems dry fruits and vegetables at relatively low temperatures (typically 30–60 °C), mitigating issues related to excessive oxidation [18]. It has received widespread attention as a means of reducing non-renewable energy consumption and pollutant emissions. At the same time, they have many advantages, including improving energy efficiency, enhancing product quality, and the ability to regulate drying temperature and air humidity [19].

The maturity of single heat pump drying technologies is reflected in their established processes, comprehensive research on heat pump components, and the development of mathematical models that accurately describe certain aspects of the drying processes for fruits and vegetables. Finally, heat pump drying fruits and vegetables offers notable advantages compared to traditional drying methods, such as air and natural drying, including higher product quality and ease of automation and control.

### 2.2. Limitations of Single Heat Pump Drying Technologies

Single heat pump drying technologies face many limitations considering the growing consumer demand for food quality and increasingly rigorous market challenges, which require urgent improvement. A feasible alternative to relying on a single energy source is to use multi-source heat pumps, which can utilize different low-temperature energy sources with different evaporators [20].

#### 2.2.1. Automation Limitations

The use of sensors is limited when using heat pump drying for fruits and vegetables, which means that temperature and humidity cannot be modified in real-time based on the drying characteristics of the material. This increases production costs and complicates the system compared with other drying methods [21]. Current numerical control technologies for single-stage heat pump drying systems focus on single-target controls, but multi-objective control systems are required [22]. Moreover, the application of adaptive adjustment systems, including artificial intelligence and fuzzy controls, remains relatively scarce, making the use of single-stage heat pump drying systems difficult for various fruits and vegetables. The automation of this technology must be improved, specifically, the accuracy of the temperature and humidity controls and the ability to adapt to the specific conditions and characteristics of fruits and vegetables. It can be predicted that whether it is the future development needs of the fruits and vegetables industry or the requirements of global sustainable development, upgrading the industrial structure and establishing a new energy-saving drying system for fruits and vegetables is important to meet human life needs and avoid unknown risks [23].

#### 2.2.2. Production Requirement Limitations

The application of sensors in single-stage heat pump drying of fruits and vegetables is often limited, preventing real-time modification of temperature and humidity based on the material’s drying characteristics. For instance, research on apple slice drying has shown that fixed parameter settings often result in uneven drying or poor energy efficiency [24]. This limitation increases production costs and complicates the system compared to alternative drying methods [21]. Current numerical control strategies for these systems predominantly focus on single-target optimization, such as maintaining constant temperature (as seen in optimization studies for banana drying [25]). However, multi-objective control systems, capable of simultaneously optimizing energy efficiency, drying rate, and product quality, are essential for practical applications [22]. Furthermore, the implementation of advanced adaptive regulation systems incorporating artificial intelligence (AI) or fuzzy logic techniques remains relatively scarce in practical drying scenarios. Studies demonstrating adaptive humidity and temperature control specifically for products like kiwifruit, for example, are still rare and primarily proof-of-concept [26]. This scarcity makes it challenging to deploy single-stage heat pump drying effectively across the diverse range of fruits and vegetables with varying drying behaviors. Consequently, enhancing the automation level of this technology, particularly improving the accuracy of temperature and humidity controls and developing adaptability to the specific drying kinetics and physical properties of different produce (as highlighted by the distinct adaptive control requirements for cherry tomatoes discussed in [27]), remains a critical research and development need.

The performance of single heat pump drying technology equipment does not meet production requirements. Most heat pump drying equipment uses air as the drying medium, which can cause oxidative damage to the dried products. A recent study proposed that using inert gas instead of air in fully enclosed circulation drying could significantly improve the quality of dried products [28]; however, research on this approach is scarce. In addition, in single heat pump systems, the compressor exhibits poor performance under high pressure, making it difficult to increase the drying scale and temperature. Drying chamber temperatures below a specific range may promote bacterial growth in dried fruits and vegetables, resulting in serious economic losses. Lee et al. [29] reported that the Coulomb force-assisted heat pump drying technology outperforms general heat pump drying in terms of drying speed and vitamin retention rate when applied to lemon slices. However, studies on heat pump drying technologies that use the Coulomb force as an auxiliary heat source in China are limited.

In summary, the performance of the equipment used in single-stage heat pump drying technology is inadequate, with notable limitations in energy consumption control, drying scale, and quality in drying fruits and vegetables.

#### 2.2.3. Challenges in Market Application

The use of single heat pump technologies in dried fruit and vegetable markets is limited. Current heat pump drying systems often rely on large-scale dryers, which involve high investment costs and are unsuitable for agricultural development in China [30]. To improve economic efficiency, fruit and vegetable heat pump dryers should be small, lightweight, and portable. Studies on seasonal processing in rural areas, such as those addressing berry preservation [27], demonstrate how such features reduce transportation costs during harvest and minimize spoilage caused by adverse weather events. Compared with their applications in biological and medical fields, manufacturing heat pump dryers for fruits and vegetables faces challenges. These include widespread issues of inconsistent equipment quality across suppliers and fragmented raw material supply chains, as reported in technical assessments of the agricultural equipment sector [31]. The product range of heat pump components is often incomplete, with inconsistent types and qualities [32]. Additionally, environmental studies highlight that refrigerant leaks in heat pump drying systems pose significant ecological risks due to high Global Warming Potential (GWP). This is particularly concerning in regions lacking strict containment protocols [33]. Moreover, refrigerant leaks in heat pump drying systems may pose severe environmental risks [34].

Overall, the high costs, poor portability, irregular product standards, and pollution risks of this technology collectively reduce its economic benefits.

#### 2.2.4. Limitations in Drying Quality

The quality of fruits and vegetables dried using single heat pump technologies must be improved. Although effective in the early drying stage, moisture redistribution within the produce during later stages significantly reduces drying speed and quality [26]. For example, the rehydration rate and nutrient retention of single-stage heat pump technologies are lower than those of microwave and freeze drying [35]. Poor drying quality may also result from insufficient research on drying characteristics. For example, drying whole lychees with single-stage heat pumps results in slower drying and a lower quality product than drying lychee pulp [36]. A previous study on heat pump drying of *Hippophae rhamnoides* (sea buckthorn) demonstrated that pretreating fruits with 2% sodium carbonate solution for 1 h achieved the highest retention of vitamins and flavonoids and the optimal color [37]. In recent years, breakthroughs in AI technology have injected new impetus into the field of fruit and vegetable drying. In the field of fruit and vegetable drying, AI technologies such as machine learning, computer vision systems (CVS), and expert systems provide innovative tools for drying modeling, process monitoring, and parameter adjustment with their powerful data mining, pattern recognition, and decision optimization capabilities [26]. For example, artificial neural network (ANN)-based models of drying kinetics accurately predict patterns of moisture transport and energy consumption [38,39]; CVS can perform online non-destructive testing on color and texture during the drying process [40]; and genetic algorithms can optimize quality attributes and process parameters [41]. These findings highlight the need to explore tailored processing methods for different types of produce.

Enhancing drying efficiency and automation requires further research into heat transfer mechanisms, nutrient changes during drying, and the development of mathematical models [42]. However, such models are still unavailable for many fruits and vegetables [43].

## 3. Combined Heat Pump Drying Technologies

**Figure 4:**
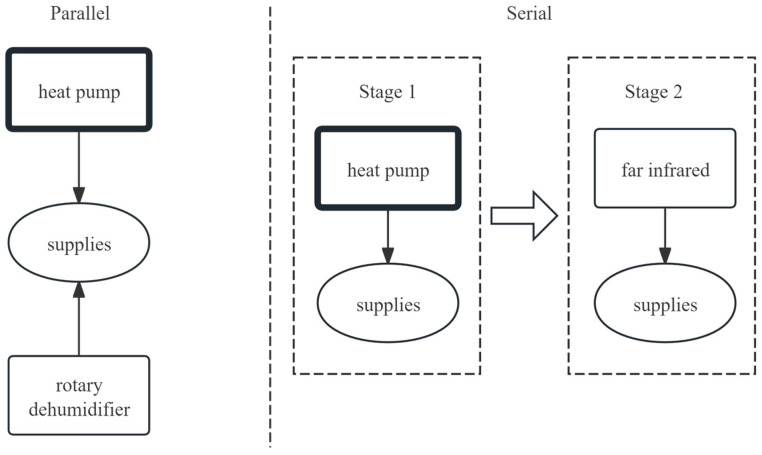
The concept comparison diagram of series vs. parallel combined drying technology.

Combining heat pump technology with other drying methods, known as combined heat pump drying, has become a key focus for fruit and vegetable drying (Figure 4). Combined systems can be classified as serial or parallel based on whether the two methods operate simultaneously [22]. Although this integrative approach significantly improves product quality, drying speed, and energy efficiency compared with those of single heat pump drying, its widespread adoption is hindered by high costs and limited applicability.

### 3.1. Principles, Configuration Selection, and Comparative Merits of Series vs. Parallel Combined Drying

Combining heat pump (HP) technology with other drying methods offers significant advantages over single HP drying, including enhanced drying efficiency, improved product quality, and reduced energy consumption. These hybrid systems are typically configured in either series (tandem) or parallel modes, each with distinct operational principles, rationales for use, selection criteria, and inherent advantages and disadvantages.

#### 3.1.1. Fundamental Principles and Rationale

The core rationale for hybrid drying lies in overcoming the limitations inherent in single drying techniques. For instance, while HP drying excels in energy efficiency and controlled low-temperature drying, it can suffer from slow drying rates, especially in the falling rate period, and potential non-uniformity. Other methods, like microwave (rapid internal heating), fluidized bed (enhanced external mass transfer), or vacuum freeze-drying (superior quality but high energy), have their own strengths and weaknesses. Hybridization aims to synergistically leverage the complementary strengths of different methods.

Series Configuration: In this approach, the drying process is divided into sequential stages. The material is processed through one drying technology (e.g., HP drying) before moving to the next (e.g., microwave drying). The output (partially dried material or conditioned air) from the first stage serves as input for the subsequent stage. This sequential operation allows for stage-specific optimization. For example, HP drying might gently remove surface moisture initially, preserving heat-sensitive nutrients, followed by a faster method like microwave to tackle bound internal water later, reducing overall time without compromising early-stage quality.

Parallel Configuration: Here, two or more drying mechanisms operate simultaneously and cooperatively on the material within the same drying chamber or stage. Their effects are concurrent and synergistic. For example, the convective airflow and dehumidification provided by the HP system work simultaneously with microwave energy penetrating the material volumetrically, or as a fluidized bed agitating the product. This simultaneous action directly addresses both external (heat/mass transfer) and internal (moisture diffusion) drying resistances concurrently, often leading to significant drying acceleration.

#### 3.1.2. Criteria for Selecting Series vs. Parallel Configuration

The choice between series and parallel configurations depends on several key factors:

Material Characteristics: The heat sensitivity, initial moisture content, size, shape, and composition (e.g., sugar content affecting glass transition) of the fruit or vegetable are critical. Materials prone to case-hardening (surface hardening trapping internal moisture) often benefit from parallel methods combining surface drying with internal heating (e.g., HP + microwave). Materials with distinct drying phases (high free water initially, bound water later) might be better suited for sequential treatment in series.

Dominant Drying Limitation: Identifying whether the drying rate is primarily limited by external conditions (low heat/mass transfer coefficients) or internal conditions (slow moisture diffusion) guides the choice. Parallel configurations excel when internal diffusion is the bottleneck, as mechanisms like microwave or electric fields can directly accelerate internal moisture movement simultaneously with convective drying. Series configurations can effectively target specific limitations in sequence.

Target Product Quality Attributes: Specific quality requirements (e.g., maximum nutrient/color retention, specific texture, high rehydration ratio) influence the choice. Some quality goals are better achieved by specific sequences (series) or synergistic actions (parallel).

Process Control Requirements: Parallel systems integrating simultaneous energy inputs (e.g., convective heat + microwave radiation) generally require more sophisticated control systems to manage interactions and prevent issues like localized overheating, compared to the potentially simpler stage-by-stage control in series systems.

Economic and Practical Considerations: Factors such as equipment cost, footprint, energy source availability (e.g., solar integration potential), scalability, and ease of material handling between stages (relevant for series) play a significant role in the selection decision.

#### 3.1.3. Advantages and Disadvantages of Each Configuration

Series configuration’s advantages: Enables precise optimization of drying conditions for each distinct stage of the drying curve (e.g., high moisture vs. low moisture content). Control within each individual stage can be relatively straightforward. It is well-suited for integrating existing dryer units into a production line and effective for processes with clear phase separations like pre-drying and finish drying.

Series configuration’s disadvantages: Requires physical transfer of material between drying stages, which can increase processing time, handling costs, and risk of product damage or contamination. The total drying time is typically the sum of the times for each stage plus transfer time. Energy recovery between physically separate stages may be less efficient than in tightly integrated parallel systems. The overall system footprint can be larger.

Parallel configuration’s advantages: Offers the potential for substantial reductions in total drying time due to the synergistic, simultaneous action of combined mechanisms. Often achieves superior drying uniformity by concurrently overcoming both surface and internal resistances. Allows for highly efficient, direct energy coupling within a single unit (e.g., HP condenser heat directly warming the drying air used for fluidization or convection). Can result in a more compact overall system design.

Parallel configuration’s disadvantages: Significantly increases system complexity and control challenges due to the interaction of multiple simultaneous energy inputs, requiring careful tuning to avoid issues like non-uniform drying or localized overheating. Initial equipment cost and complexity are generally higher due to the integration of diverse technologies. Parameter optimization for the interacting mechanisms can be intricate. Effectiveness might be constrained by material properties like thickness, particularly for penetration-limited methods like microwaves or infrared.

Understanding these fundamental principles, selection criteria, and comparative merits is essential for designing and implementing effective combined heat pump drying systems tailored to the specific requirements of various fruits and vegetables. The following sections detail specific examples of prevalent parallel (Section 3.2) and series (Section 3.3) combinations employed in the field.

### 3.2. Combined Heat Pump Drying Technology in Parallel

Parallel connection refers to the heat pump and other drying units operating independently but working in concert, acting together in the same drying stage, complementing each other’s advantages.

Combined parallel heat pump drying methods integrate heat pump technologies with large-scale drying methods from other fields, such as dehumidification drying wheels, fluidized beds, vacuum freezing, and high-voltage electric field drying methods. These combinations enhance the advantages of single heat pump drying.

#### 3.2.1. Heat Pump Combined with Fluidized Bed Drying

Heat pump drying combined with the fluidized bed method uses exhaust gas recycling from fluidized bed devices to reduce energy consumption, resulting in a strong market potential [44]. Single heat pump drying systems typically use box structures that cannot support continuous, long-term drying. Traditional fluidized bed drying operates at high temperatures with high energy consumption, making it unsuitable for processing heat-sensitive materials such as fruits and vegetables [45]. Zhang et al. [46] conducted carrot drying tests using a fluidized bed heat pump drying system, and the moisture content decreased from nearly 100% to <10% within 10 h. Zielinska et al. [47] combined a heat pump, fluidized bed, vacuum freezing, and microwave drying. This system reduced shrinkage and scorching rates when applied to peas while increasing drying speed. Combining a heat pump with vacuum freeze-drying yields high-quality results similar to those obtained with pure vacuum freeze-drying but with shorter drying time and lower energy use [48]. Wang et al. [49] dried king oyster mushrooms using a combination of dehumidification drying wheels and heat pump technology. This system performed well at high humidity, overcoming heat pump limitations (i.e., low temperature and low humidity only) and reducing energy use compared with that of standalone wheel drying. Wang et al. [50] dried Chinese yam using a high-voltage electric field and heat pump. Han et al. [51] studied the effects of vacuum drying (VD) and heat pump drying (HPD) together on the drying characteristics and physicochemical properties of pineapple. The sample treated with VD5h + HPD5.5h had the highest brightness and the smallest color difference, and its volume shrinkage and rehydration ability were significantly better than other drying methods. The VD5h + HPD5.5h method has been proven to be the best method for drying pineapple. Compared with single heat pump drying, this method reduced the drying time, enhanced efficiency, and maintained quality.

**Figure 5:**
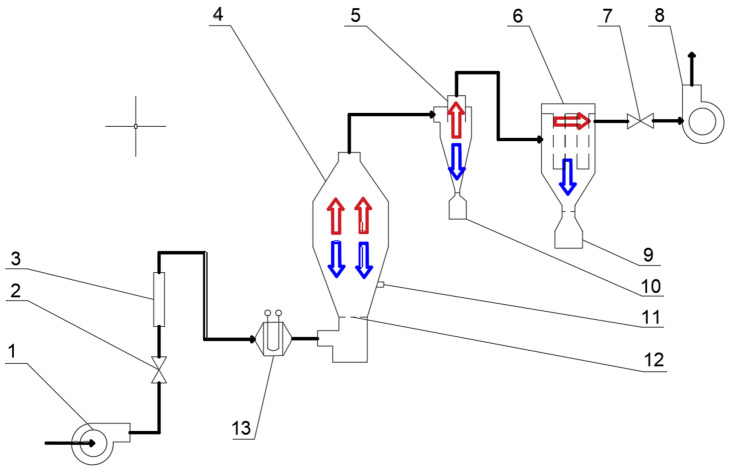
Schematic diagram of heat pump fluidized bed drying [52]. (1) Blower; (2,7) Regulating valves; (3) Gas rotameter; (4) Fluidized bed dryer; (5) Cyclone separator; (6) Bag filter; (8) Induced draft fan; (9,10) Collection tank; (11) Sampling port; (12) Gas distribution plate; (13) Electric heater. Notes: The red arrows indicate the direction of hot air flow, while the blue arrows represent the direction of cold air flow.

Parallel combined drying systems that integrate heat pump and fluidized bed technologies exhibit significant advantages in energy saving and efficiency for fruit and vegetable processing. Heat pump drying enables energy recycling by recovering latent heat and sensible heat in the exhaust gas, whereas fluidized bed drying improves heat and mass transfer through fluidization. Combining these two approaches provides an innovative solution for low-temperature and high-efficiency drying (Figure 5). Shi et al. [44] studied fluidized bed drying with a closed-circuit circulation heat pump. And it was proposed that the use of partial exhaust gas circulation could significantly improve the dehumidification energy consumption ratio (SMER). The simulation results demonstrated that when the evaporation and condensation temperatures were 20 °C and 75 °C, respectively, increasing the circulation ratio increased the SMER to 2.8 kg/(kW-h), representing an energy-saving improvement of approximately 40% compared with that of the conventional system. This mechanism efficiently reduces drying energy consumption and avoids exhaust gas emissions, which aligns with environmental sustainability considerations.

The unique advantage of fluidized bed drying is that it reduces the material surface boundary layer resistance through gas–solid fluidization, accelerating moisture migration. Zhu et al. [52] developed a heat pump fluidized bed dryer and tested it using diced carrots. They found that the tumbling of materials in the fluidized state significantly improved the heat transfer efficiency, reducing the drying time by >30% compared with that of a traditional box heat pump. Moreover, the water content of the diced carrots was reduced from 90% to 10% in only 9 h, and the drying uniformity was superior to that obtained through flow drying, confirming the central role of the fluidized bed in enhancing drying intensity. Tellab et al. [53] Research has shown that using the weight method to rehydrate soybeans to 22% (dry basis) and drying them in a fluidized bed dryer at 40 °C can achieve the highest drying rate (98%), the highest germination rate (88%), and the lowest grain cracking rate (7%), which is the optimal solution for minimizing losses. Rajendran et al. [54] found that fluidized bed treatment and addition of energy carriers reduced the drying time of turkey berries by 12 times and 10 times, respectively. The fluidized bed pretreatment method achieved the fastest drying process and was completed within 330 min. In addition, compared to untreated samples, a minimum shrinkage rate of approximately 39%, tolerable color changes, and maximum retention rate of vitamin C were achieved.

The intermittent drying strategy optimizes the comprehensive performance of the heat pump fluidized bed parallel system. Liu et al. [55] discovered that for wheat drying, intermittent drying with a heat pump-fluidized bed improved energy utilization by 15% compared with that of continuous drying while reducing the wheat germination rate to <5%. Intermittent operation, achieved by periodically switching the hot air on and off, could alleviate local overheating issues caused by continuous drying, especially when processing heat-sensitive fruits and vegetables, thereby effectively retaining the color and nutrients. Liu et al. also indicated that the optimal selection of intermittent ratio η is key to balancing drying efficiency and quality. The system’s thermal efficiency reached 93.75% when η was equal to 2/3, providing a reference for optimizing fruit and vegetable drying parameters.

In summary, the parallel combination of the heat pump and fluidized bed drying system achieves the dual goals of low-temperature, high-efficiency drying, energy-saving, and environmental friendliness through closed-circuit energy recovery, fluidized enhanced mass transfer, and intermittent operation regulation. Future research should focus on developing multi-stage fluidized bed structure designs and intelligent control strategies to better adapt to the drying characteristics of different fruits and vegetables, thereby facilitating the large-scale application of this technology in the food industry.

#### 3.2.2. Heat Pump Combined with Dehumidification Drying

Han et al. [56] used white radish slices to test the synergy of heat recovery and humidity control when integrating heat pump circulation and dehumidification drying methods in parallel. When the drying temperature increased from 35 °C to 45 °C, the joint drying method enhanced the maximum drying rate by 60.9%, reduced the drying time by 2.5 h, and increased the rehydration rate by 6% compared with those of single heat pump drying. Wu et al. [57] also reported a heat pump system that uses the evaporator to absorb ambient heat, which is warmed up by the compressor and then released into the drying chamber through the condenser. Simultaneously, a low-temperature regeneration rotor dehumidification device uses polymer adsorbent materials to dehumidify the circulating air, thereby reducing the relative humidity of the drying medium. The authors applied this joint drying system to carrot slices and demonstrated that the effective moisture diffusion coefficient at 45 °C reached 6.47 × 10^−10^ m^2^/s, which was 1.18 times higher than that achieved with a single heat pump. Moreover, the unit energy consumption was reduced by 39.46%. Sun et al. [58] studied the combination of closed heat pump drying system and rotary dehumidification (HPRD). The results showed that HPRD reduced the drying time of banana slices during low-temperature processing, while retaining more color and vitamin C content than a closed heat pump drying system (THPS). The drying rate of banana slices dried at low temperatures increased by 41%. In addition, it increased the average vitamin C content by 12% and reduced the total color difference by an average of 16%, which is 26% higher than the water extraction rate.

**Figure 6:**
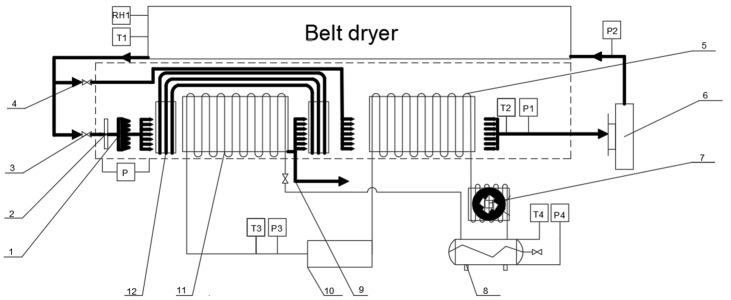
Diagram of a heat pump dehumidifying drying device [59]. (1) Heater; (2) Heat exchanger; (3,4) Regulating valves; (5) Condenser; (6) Feed conveyor belt drive motor; (7) Dehumidifying fan; (8) Compressor; (9) Expansion valve; (10) Evaporator; (11) Condenser; (12) Condensation pipe; RH1 = Feed inlet; T1, T2, T3, and T4 = temperature sensors; P, P1, P2, P3, and P4 = pressure sensors.

The parallel operation of the heat pump, dehumidification, and drying (Figure 6) could create a high-temperature and low-humidity drying environment, thereby reducing heat loss and improving the moisture migration drive. For example, Sun et al. [59] demonstrated that dehumidification drying systems significantly reduce microbial activity through internal circulation dewatering and precise temperature control, extending the shelf life of dried carrot products by >1 year. By comparing six types of fruits and vegetables, the authors observed that vitamin C retention after dehumidification drying exceeded 90% of the fresh state, and the sensory scores, particularly for color and rehydration, were significantly better than those obtained with hot air drying.

Parallel combined drying technology offers energy-saving benefits and quality preservation, especially for heat-sensitive fruits and vegetables, demonstrating considerable market potential. In summary, parallel heat pump dehumidification combined with drying technology, achieved through precise temperature and humidity control, provides a highly efficient and environmentally friendly solution for fruit and vegetable processing. With further process optimization and equipment innovation, this technology is expected to be applied on a large scale in the processing of agricultural products.

#### 3.2.3. Heat Pump Combined with Vacuum Freeze-Drying

Parallel heat pump–vacuum freeze drying realizes efficient energy circulation by using a heat pump’s cooling and heating functions in stages, thereby reducing switching loss between cold and heat sources compared with that of traditional freeze drying. Song et al. [60] demonstrated that vacuum freeze drying of Jingning apples preserves the shape and internal structure of the dried fruit, better meeting consumer preferences in terms of appearance and taste. However, as the vacuum freeze-drying process involves multiple phase transitions, the issue of excessive energy consumption should be considered. In contrast, owing to its working principle of recycling circulating environmental heat, heat pump drying offers a shorter drying time and lower energy consumption than vacuum freeze drying. Zheng [61] proposed that vacuum freeze drying could be combined with other drying methods to reduce energy consumption; specifically, vacuum freeze–heat pump combined drying was identified as a promising developmental direction. Cheng et al. [62] reported that using the combined heat pump–vacuum freeze-drying technology to dry mushrooms could reduce energy consumption by 37.69% compared with that of vacuum freeze-drying alone. Combined heat pump–vacuum freeze-drying technology can achieve a product quality comparable to that of vacuum freeze-drying while significantly reducing energy consumption. Therefore, this approach represents an important technological alternative to traditional vacuum freeze drying. Notably, although the combined drying method offers both quality retention and energy-saving advantages, its practical application in the food processing field in China remains in the exploratory stage.

#### 3.2.4. Heat Pump Combined with High-Voltage Electric Field Drying

**Figure 7:**
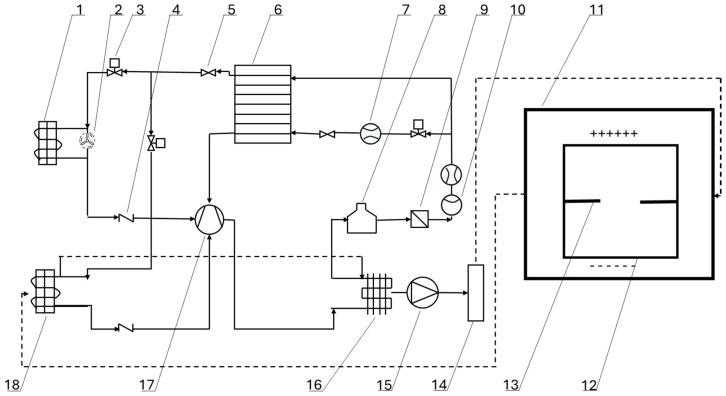
Schematic diagram of a high-pressure electric field–heat pump combined drying system [65]. (1) Outdoor evaporator; (2) External fan; (3) Solenoid valve; (4) Check valve; (5) Electronic expansion valve; (6) Economizer; (7) Mass flowmeter; (8) Liquid receiver; (9) Drying filter; (10) Sight glass; (11) Drying chamber; (12) High-voltage electric field plate; (13) Material tray; (14) Electric heating; (15) Circulating fan; (16) Condenser; (17) Compressor; (18) Indoor evaporator.

Parallel heat pump–high-voltage electric field combined drying is a staged, synergistic, energy-saving technology (Figure 7). The heat pump provides warm air for initial dehydration, reducing surface moisture from the material. In turn, this enables a high-voltage electric field for electric penetration, which accelerates the migration of internal moisture to the surface and its subsequent evaporation. These two phases, performed in alternating or sequential operation, preserve the energy efficiency advantages of the heat pump. They also overcome the mass transfer bottleneck of traditional drying, significantly reducing the consumption of time and energy and protecting the heat-sensitive active ingredients. Wang et al. [63] demonstrated that high-voltage electric field drying technology can preserve the color and active ingredients of herbs while being energy efficient. Meng et al. [64] found that high-voltage electric field drying technology shortens the drying time of carrots, improves the effective water diffusion rate (D-Eve) and specific water extraction rate (SMER). However, while this technology holds considerable potential, it requires further improvements, particularly in energy optimization. Wang et al. [65] used banana chips to study high-voltage electric field–heat pump combined drying technology. They demonstrated that the method significantly improved the drying rate, reduced energy consumption, and improved quality (e.g., color and luster) compared with those of single heat pump drying. The drying rate was positively correlated with heat pump temperature and electrical field intensity and negatively correlated with slide thickness and overall drying speed. Additionally, the moisture diffusion coefficient increased with increasing temperature and electric field and decreased with increasing thickness. Moreover, the Page model accurately described the drying process and verified the high efficiency and energy savings of combined drying. Although this technology can effectively optimize the drying process, further investigation into the electrode parameters, influence of multifactor coupling, and retention rate of active ingredients is required to determine the optimal conditions. Such research will provide theoretical support for reducing energy when drying fruits and vegetables while improving quality.

### 3.3. Combined Heat Pump Drying Technology in Series

Tandem means that the heat pump works in succession with other technologies in stages to form a multistage drying process.

#### 3.3.1. Heat Pump Combined with Hot Air Drying

The most common series system combines a heat pump with a hot air dryer. The heat pump absorbs heat from low-temperature sources (e.g., air or water) and converts it into high-temperature heat for warm air drying. In series systems, exhaust air passes through the evaporator of the heat pump for cooling and dehumidification. Waste heat is recovered, reheated via the condenser, and reused for drying, achieving efficient energy recycling. Compared with standalone hot air or heat pump drying, the combined method improves both drying quality and speed. Zhang et al. [46] tested hot air, heat pump, and combined drying on green peppers and carrots, demonstrating that the combined method exhibited better rehydration rates and less shell shrinkage. Notably, the use of heat pump drying in the early stages saves energy, while switching to hot air drying during later stages maintains drying speed and effectiveness, combining the advantages of both methods.

#### 3.3.2. Heat Pump Combined with Far-Infrared Drying

**Figure 8:**
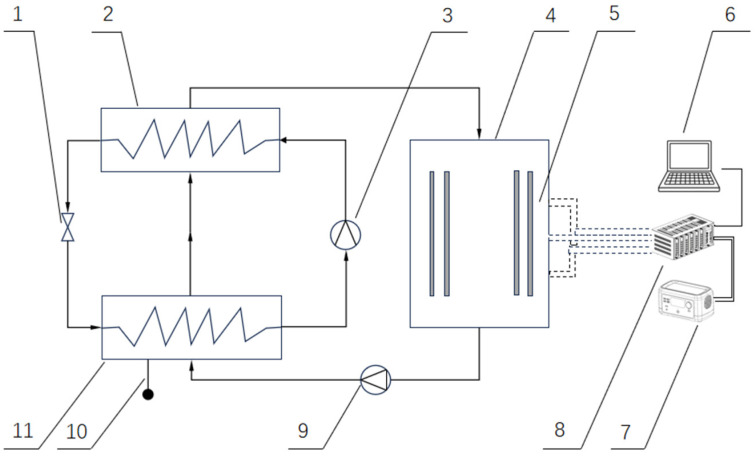
Schematic diagram of a combined far-infrared heat pump drying device [73]. (1) Throttle valve; (2) Condenser; (3) Compressor; (4) Drying chamber; (5) Far-infrared heater; (6) Computer; (7) DC regulated power supply; (8) Data acquisition module; (9) Circulating fan; (10) Drain; (11) Evaporator.

Far-infrared, microwave, and solar energy technologies have also been used in fruit and vegetable drying. While heat pump drying enables energy savings and offers environmental benefits, it can result in uneven drying. To address this limitation, researchers have focused on far-infrared technology combined with heat pump drying to improve the stability of environmental ground temperature and humidity during the drying process (Figure 8). The use of far-infrared technology-assisted heat pump drying (far-infrared radiation combined heat pump drying, FIR-HPD) materials can solve the single heat pump’s uneven heating problems, thereby improving drying efficiency [66]. Bai et al. studied the effects of far-infrared technology-assisted heat pump (FIR-HPD) on the quality, volatile flavor compounds, and antioxidant activity of Xiaomi pepper. In terms of physical properties, FIR-HPD had the highest drying rate, shortest drying time, better color, and higher hardness. In terms of flavor, FIR-HPD can retain more ester compounds [67]. For example, Yang et al. [68] summarized the application of heat pump technology combined with far-infrared drying technology in longan, banana, garlic, other fruits, and vegetables. They indicated that far-infrared assistance provides a good solution to the problem of non-uniformity of material drying while also offering high drying efficiency, minimal heat loss, and a wide radiation spectral range. Far-infrared technology primarily uses radiation in the 3–1000 μm range, transmitting infrared heat energy into the material without the need for a medium [69]. During the drying process, the surface of the material is dried through continuous heat absorption. As the far-infrared radiation energy matches the vibration frequency of water molecules, it stimulates the resonance of water molecules within the fruit, thereby accelerating their migration [70]. Far-infrared heating also has a small thermal inertia and moderate penetration ability [71]. Therefore, the drying material is heated from the inside out, which addresses the issue of uneven drying commonly observed in the later stages of heat pump drying [16]. In contrast, infrared radiation heating and drying alone produce water vapor that is difficult to eliminate, affecting the experimental results. Therefore, the combination of heat pumps and far-infrared drying offers complementary advantages, as confirmed by numerous studies.

Currently, far-infrared-assisted heat pump drying systems are limited by their high initial cost and difficult technical integration. Therefore, advancements in materials science and intelligent control technologies are needed to facilitate the large-scale application of this technology.

#### 3.3.3. Heat Pump Combined with Microwave Drying

**Figure 9:**
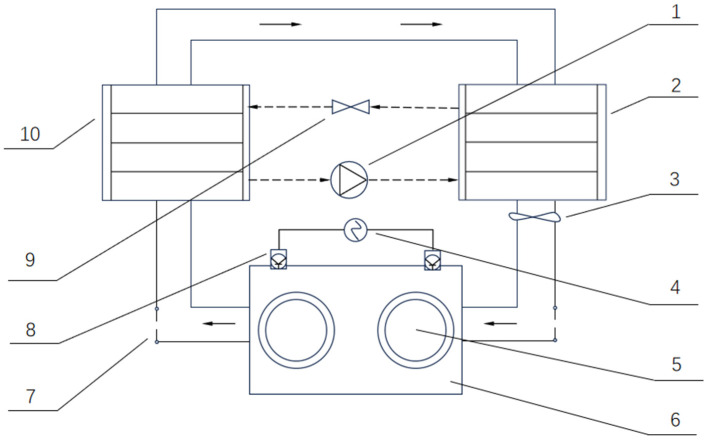
Schematic diagram of a heat pump–microwave combined drying device [75]. (1) Compressor; (2) Condenser; (3) Fan; (4) Microwave power supply; (5) Water bag mounting position; (6) Drying chamber; (7) Dampers; (8) Microwave generator; (9) Throttle; (10) Evaporator.

Combined microwave and heat pump drying technology (Figure 9) offers two main advantages. First, microwave drying technology uses the moisture within the material to absorb microwave energy. Second, the microwave effect generates molecular vibration and internal heating, causing a temperature gradient with high internal temperature and low external temperature, thereby significantly accelerating the drying rate [72]. In heat pump drying, the air mass transfer coefficient is relatively low, which significantly reduces the drying rate and increases energy consumption. Notably, microwave drying can address these issues. Microwaves are high-frequency electromagnetic waves; under the action of a magnetic field, molecules within the material constantly move, generating internal friction that produces heat energy for drying [73]. Microwaves can penetrate thick materials, resulting in uniform internal and external drying. Moreover, owing to the high energy conversion rate of this technology, the direction of heat transfer from both the interior and surface is consistent with the direction of water evaporation. This alignment results in outstanding energy during microwave heating. Given the distinctive characteristics of microwave drying, it is generally combined with other drying technologies to improve the efficiency and operational costs of the drying process [74]. For example, microwave drying alone carries the risk of a large temperature gradient and thermal runaway; however, combining it with heat pump drying technology offers a complementary approach. Chien et al. [75] used a combination of heat pump and microwave technologies to dry apple slices and compared the results with products dried by each technology alone. The authors demonstrated that products dried using the combined drying method exhibited the best appearance and highest vitamin retention. Moreover, microwave sterilization and disinfection, along with their rapid drying speed, effectively address the issues of bacterial growth and slow drying speed during the later stages of heat pump drying. Although microwave drying alone can cause localized hardening due to ingredient loss, this issue is addressed when microwave drying is combined with heat pump drying. However, microwave–heat pump joint drying technology faces several challenges, including high equipment purchase and maintenance costs, poor technology control, and concerns regarding compatibility with complex materials and suitability. These issues can be addressed through advancements in modular design and intelligent control technology, allowing for wider adoption of this drying technology.

#### 3.3.4. Solar-Assisted Heat Pump Drying

**Figure 10:**
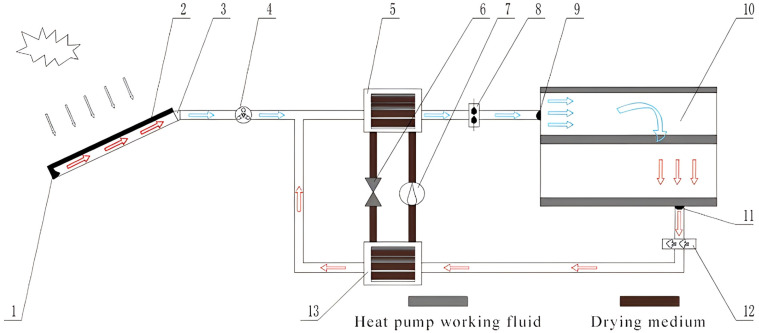
Solar-assisted heat pump drying system [76]. (1) Air intake; (2) Heat collector; (3) Air intake duct; (4) Centrifugal fan; (5) Condenser; (6) Throttle valve; (7) Compressor; (8) Supply fan; (9) Air inlet; (10) Drying room; (11) Air outlet; (12) Exhaust fan; (13) Evaporator.

**Figure 11:**
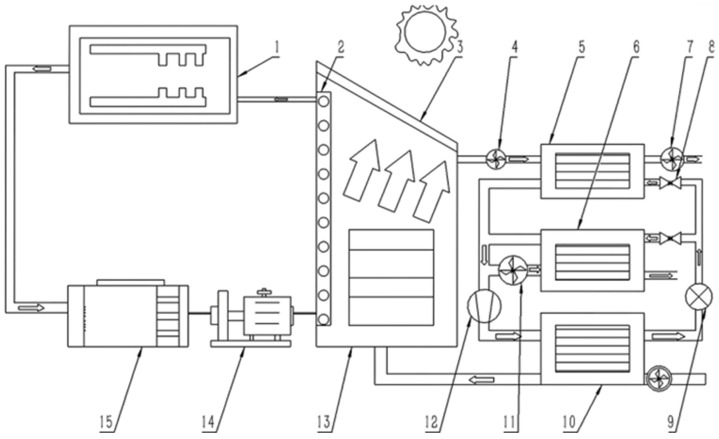
Schematic diagram of a solar–air heat source drying system [79]. (1) Evacuated tube collector; (2) Hot water radiation panel; (3) Metal absorption plate; (4) Bypass exhaust fan; (5) Evaporator no. 1; (6) Dryer no. 2; (7) Exhaust fan; (8) Solenoid valve; (9) Expansion valve; (10) Condenser; (11) Induced draft fan; (12) Compressor; (13) Drying chamber; (14) Water pump; (15) Water storage pipe.

**Figure 12:**
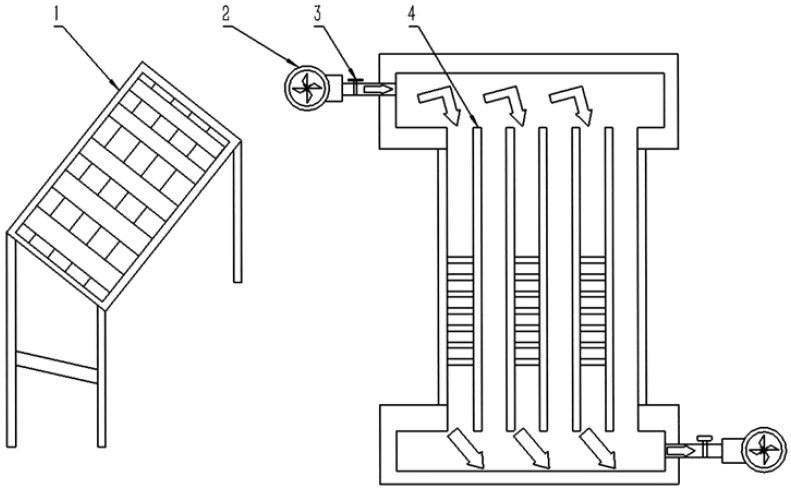
New double-pass vacuum tube solar air collector [82]. (1) New-type collector; (2) Induced draft fan; (3) Flow meter; (4) Vacuum double-pass glass tube with a heat absorption coating.

Solar energy is a clean energy source that has received global attention, resulting in its rapid development. For example, Zhang et al. [76] integrated solar and heat pump technologies, including solar-assisted heat pump drying, double-pass vacuum solar air collector systems, and solar–air source heat pump drying systems, to dry wood. However, solar energy is greatly affected by the weather and the number of daily sunshine hours, which differ across China, resulting in variable performances. Solar-assisted heat pump drying systems (Figure 10) are divided into direct expansion, indirect expansion, and combined direct and indirect expansion types [77]. Users can select the system that best suits their specific needs, thereby reducing energy consumption and optimizing the drying process [78]. Solar–air heat source drying systems use both air and solar water source evaporators to recycle heat energy (Figure 11). This design ensures that the internal temperature of the drying system fluctuates within a reasonable range, thereby improving the drying effect and reducing the environmental burden [79]. The newly developed double-pass vacuum tube solar air collector system (Figure 12) uses air as the medium, which reduces the number of intermediate steps and improves the heat exchange efficiency of the system. With its simple structure and large air output from the collector, this system can reduce heating costs and improve the drying effect. Moreover, this system addresses the problems of scale formation and corrosion associated with traditional solar energy, considerably improving the lifespan of the system and reducing maintenance costs [80]. In terms of energy consumption, Qin et al. [81] reported that the use of solar–heat pumps to dry fruits and vegetables saved 150% and 65% of energy compared with that of hot air and vacuum freeze drying, respectively. Moreover, Shen et al. [82] investigated the use of heat pump technology combined with solar technology to dry walnuts. Their orthogonal experiments demonstrated that this combined approach significantly reduced the drying cycle. Mortezapour et al. [83] mixed photovoltaic solar energy and heat pump drying technology, achieving drying rates of at least 70%. Rulazi et al. [84] studied the drying and analysis of tomatoes and carrots using solar-assisted heat pump drying technology (SAHPD) to determine their thermal and economic performance. The results showed that the initial moisture content of tomatoes (Lycopersicum esculentum) and carrots (Daucus carota) decreased from 93% and 88% to 10% within 11 and 12 h, respectively. The coefficient of performance (COP), drying time (DT), specific moisture extraction rate (SMER), and thermal efficiency (TE) are 3.4, 2.3 kg/h, 1.33 kg/kWh, and 54.0%, respectively. The solar-assisted heat pump drying technology (SAHPD) has both technical and economic feasibility in tomato and carrot drying and has the potential for promotion.

#### 3.3.5. Ultrasound-Enhanced Heat Pump Drying

Direct-contact ultrasound, delivered through an ultrasonic radiation disk, transfers energy directly into the raw material. This causes expansion and movement within its internal structure, reducing airflow resistance and promoting the movement and internal diffusion of moisture, thereby accelerating the drying process [85]. This approach reduces drying time and increases the drying rate by approximately 20–40% [86]. Direct-touch ultrasonic drying does not require high temperatures, avoiding the loss of heat-sensitive nutrients [87]. This approach also reduces the collapse of raw material tissues, improving the taste and quality [88]. However, the effectiveness of direct-touch ultrasonic drying is significantly reduced for raw materials thicker than 5 cm [89]. The system also requires a high initial investment of approximately 35% more than that for traditional drying techniques [90], as well as precise power control to prevent localized overheating [91]. Xue et al. [92] used direct-contact ultrasound and heat pump technology for the joint drying of fruits and vegetables to investigate ultrasound-enhanced heat pump drying (CU-HPD). They found that direct-contact ultrasound accelerated the mass transfer rate of fruits and vegetables while drying. Combining the two methods could improve heat pump drying and heat transfer bias toward the surface, addressing the issue of insufficient drying near the surface. The research results of Wang et al. [93] showed that ultrasound has a significant strengthening effect on the drying and dehydration process of HPD. Higher drying temperature and ultrasound power can lead to an increase in micropores in carrot slices, as well as an increase in the content of polyphenols, flavonoids, and niacin. The reinforcement effect of ultrasound on HPD process can significantly improve the dehydration rate and enhance product quality.

Ultrasound-enhanced heat pump drying technologies combine the advantages of direct-contact ultrasound and heat pump drying systems. The heat pump recovers heat energy, reducing energy loss, while ultrasound reduces the drying time of the raw materials. Combining these two systems reduces energy consumption by >30% compared with that of traditional hot-air drying systems. When using a single heat system, the stable temperature hardens the surfaces of the raw materials, which is not conducive to the dissipation of internal moisture. By combining with ultrasonic drying, the internal loosening of the raw material promotes moisture diffusion from the interior to the surface, preventing surface hardening. This leads to more uniform drying of the raw material and an improved overall drying effect [94]. Given the effective combination of these two technologies, the application of the system is being actively expanded beyond its original intended uses. The proposed system has been successfully applied to apples, carrots, and other high-sugar fruits and vegetables, reducing the duration of the drying cycle by 25% and improving the retention rate of vitamin C by 15% [95]. These effects improve the quality of fruits and vegetables, reduce production costs, and increase efficiency [96]. Liu et al. [97] investigated the effects of ultrasound (US) pretreatment, heat pump drying temperature, and drying time on the moisture state and viscoelasticity of scallop adductor muscle, as well as their correlation. As the drying process progresses, the transverse relaxation time (T2) remains relatively stable, while T2 shifts to the left with the increase in US pretreatment and drying temperature for free water and fixed water. The creep compliance increases, but with the increase in US pretreatment and drying temperature, the relaxation modulus decreases. However, the creep compliance shows a decreasing trend, but with the progress of drying, the relaxation modulus shows an increasing trend; that is, the moisture content decreases. In the future, the coupling parameters of CU-HPD should be further optimized, and a low-cost ultrasonic device should be developed to promote its industrialization.

The current problems of ultrasound-assisted heat pump drying technology and the corresponding measures are summarized as follows: in the process of ultrasound-assisted heat pump drying, the continuous work of ultrasonic equipment leads to a gradual increase in the temperature of the transducer device, which will affect the normal work of the equipment if it is not dissipated in a timely manner, thus affecting the drying rate and quality of agricultural products. Therefore, in order to reduce the heat generated, future research needs to consider the development of an efficient and applicable ultrasonic transducer cooling system, thereby enhancing the effect of ultrasound-assisted heat pump drying technology in practical applications, and providing strong support for its input into the actual production [98].

## 4. Conclusions

Heat pump technologies used for fruit and vegetable drying are generally classified as single or combined heat pump-based systems, with the latter generally outperforming the former. Improvements to standalone heat pump systems should focus on core components such as compressors and drying media. For hybrid systems, future research should optimize transition points between drying technologies, explore multi-technology integration (e.g., serial or parallel combinations), and advance adaptive control systems to develop cost-effective, specialized equipment. Both approaches require enhanced automation and tailored solutions to overcome barriers to market adoption.

Fruit and vegetable drying requirements constantly evolve to meet diverse demands, including quality, production timelines, and esthetic standards [85]. However, current heat pump technologies (single or hybrid) do not generally satisfy all these criteria simultaneously. To enhance drying efficacy, scalability, and alignment with consumer expectations and sustainable development goals, researchers must refine hybrid technologies, upgrade standalone systems, and investigate the mechanical and thermodynamic behaviors of various products during the drying process. In addition, reducing equipment costs, accelerating drying rates, improving product quality, and minimizing energy consumption are critical to commercializing heat pump-based drying systems and ensuring their adaptability to industry trends.

## Data Availability

The datasets generated for this study are available upon request from the corresponding author.
